# A metallic mosaic phase and the origin of Mott-insulating state in 1T-TaS_2_

**DOI:** 10.1038/ncomms10956

**Published:** 2016-03-10

**Authors:** Liguo Ma, Cun Ye, Yijun Yu, Xiu Fang Lu, Xiaohai Niu, Sejoong Kim, Donglai Feng, David Tománek, Young-Woo Son, Xian Hui Chen, Yuanbo Zhang

**Affiliations:** 1 State Key Laboratory of Surface Physics and Department of Physics, Fudan University, Shanghai 200433, China; 2 Collaborative Innovation Center of Advanced Microstructures, Nanjing 210093, China; 3 Hefei National Laboratory for Physical Science at Microscale and Department of Physics, University of Science and Technology of China, Hefei, Anhui 230026, China; 4 Key Laboratory of Strongly Coupled Quantum Matter Physics, Chinese Academy of Sciences, School of Physical Sciences, University of Science and Technology of China, Hefei 230026, China; 5 Korea Institute for Advanced Study, Hoegiro 85, Seoul 02455, Korea; 6 Physics and Astronomy Department, Michigan State University, East Lansing, Michigan 48824, USA

## Abstract

Electron–electron and electron–phonon interactions are two major driving forces that stabilize various charge-ordered phases of matter. In layered compound 1T-TaS_2_, the intricate interplay between the two generates a Mott-insulating ground state with a peculiar charge-density-wave (CDW) order. The delicate balance also makes it possible to use external perturbations to create and manipulate novel phases in this material. Here, we study a mosaic CDW phase induced by voltage pulses, and find that the new phase exhibits electronic structures entirely different from that of the original Mott ground state. The mosaic phase consists of nanometre-sized domains characterized by well-defined phase shifts of the CDW order parameter in the topmost layer, and by altered stacking relative to the layers underneath. We discover that the nature of the new phase is dictated by the stacking order, and our results shed fresh light on the origin of the Mott phase in 1T-TaS_2_.

## Abstract (web summary)

In correlated materials, new phases emerge when the balance between many-body interactions is perturbed. Here, Ma *et al*. induce a mosaic charge-density-wave phase out of Mott insulating state in layered 1T-TaS_2_ by voltage pulses, which reveals a dominating role of interlayer stacking order.

## Graphical abstract

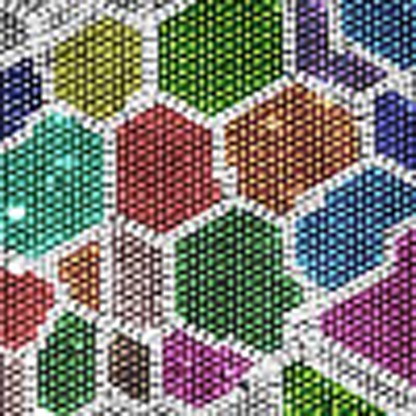

When the correlation between electrons become predominant, the interaction may lead to the localization of electrons in materials with half-filled energy bands, and turn the otherwise metallic systems into insulators^1^. Such insulator (the so called Mott insulator, MI), therefore, serves as an ideal starting point for the study of strongly correlated electron systems. Indeed, MI and its transition to metallic state (commonly referred to as metal–insulator transition) form the basis of our understanding of various magnetic phenomena^2^,^3^ and high-temperature superconductivity^2^,^4^,^5^.

The correlation effects play an important role in layered transition metal dichalcogenide 1T-TaS_2_, which is believed to turn into a MI after a series of charge-density-wave (CDW) phase transitions as the temperature is lowered^6^,^7^,^8^,^9^,^10^. The insulating ground state of 1T-TaS_2_, however, differs from typical MIs in that it resides inside a commensurate CDW (CCDW) state. As a result the localization centres in 1T-TaS_2_ are CDW superlattices, instead of atomic sites found in conventional MIs; there is also no apparent magnetic ordering accompanying the insulating ground state in 1T-TaS_2_ (refs 8, 11, 12, 13). Meanwhile, because of the close proximity of the various competing charge-ordered phases in energy, external perturbations can effectively modulate the CCDW (and thus the Mott phase) in 1T-TaS_2_ and induce a myriad of phase transitions^14^,^15^,^16^,^17^,^18^,^19^,^20^,^21^,^22^,^23^,^24^,^25^,^26^,^27^,^28^,^29^,^30^. 1T-TaS_2_ is therefore well suited to be a test bed for MI and other related strongly correlated phases.

In this study, we use voltage pulses from the tip of a scanning tunnelling microscope (STM) to create a mosaic CDW state out of the insulating ground state of 1T-TaS_2_ following a procedure described in refs 18, 31. We found that the mosaic state exhibits a metallic behaviour that is fundamentally different from the parent insulating state. Atomically resolved mapping of the mosaic metallic (MM) phase uncovers the root of such difference: each domain in the top layer of the mosaic phase is characterized by well-defined phase shift of the CDW order parameter with respect to neighbouring domains, and to the layer underneath; the altered stacking of CDW superlattice dictates whether the new phase is insulating or MM phase. Our results therefore provide fresh insight to the origin of the insulating ground state in 1T-TaS_2_ which has so far been shrouded in controversies^32^,^33^,^34^. Moreover, we find that the MM phase created at low temperature is metastable in nature: it switches back into the insulating phase after a thermal cycle. Such observation links the MM phase to the metastable phases of 1T-TaS_2_ induced by ultra-fast laser pulses^24^ and current excitation^25^,^28^,^29^,^30^. Our result may therefore provide a microscopic understanding for those novel phases that are of importance in practical applications.

## Results

### Voltage-pulse-induced MM phase

**Figure 1:**
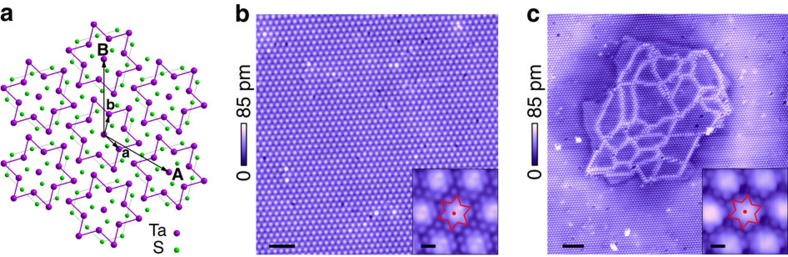
MM state induced from CCDW ground state in 1T-TaS2 by voltage pulses. (**a**) Schematic of a monolayer 1T-TaS_2_ crystal viewed from top. Only the topmost layer of S atoms are shown here for clarity. The interlocked clusters of Ta atoms (the David-star) in the CCDW state are sketched in purple, and the grey lines outline the S atoms accompanying each David-star. **A** and **B** are in-plane basis vectors of the CCDW superlattice, whereas **a** and **b** are the basis of the underlying atomic lattice. (**b**) STM topography of the cleaved surface of pristine 1T-TaS_2_. The 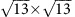 triangular CCDW superlattice is resolved at 6.5 K. Scale bar, 5 nm. Inset: a zoomed-in view of the CCDW order. Individual S atoms are resolved as small blobs on each CDW cluster, which enable us to locate the Ta atoms underneath in David-star formation (red). Scale bar, 0.5 nm. (**c**) Topographical image of an MM patch generated by a 2.8-V voltage pulse in a MI background. Scale bar, 10 nm. Inset: CCDW charge order of the MM state with the same David-star formation. Scale bar, 0.5 nm. STM images were taken under the tunnelling condition *V*_t_=0.5 V and *I*_t_=0.1 nA (main panels), or *V*_t_=0.04 V and *I*_t_=2 nA (insets).

1T-TaS_2_ bulk crystal has a layered structure, with each unit layer composed of a triangular lattice of Ta atoms, sandwiched by S atoms in an octahedral coordination. The Ta lattice is susceptible to in-plane David-star deformation where 12 Ta atoms contract towards a central Ta site^6^ as illustrated in Fig. 1a. Below 180 K the crystal enters a CCDW phase, where the David-stars become fully interlocked, forming a 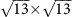 superlattice^8^. Such commensurate lattice modulation is accompanied by electronic reconstructions, which split the Ta 5*d*-band into several submanifolds, leaving exactly one conduction electron per David-star. Strong electron–electron interaction further localize these electrons, and leads to an insulating ground state^8^.

Displayed in Fig. 1b is the surface of 1T-TaS_2_ in the MI-CCDW phase imaged by STM at 6.5 K. A prominent CCDW superlattice is clearly resolved with each of the bright spots corresponding to a CDW cluster. Close examination of individual cluster reveals the position of the S atoms in the topmost layer (Fig. 1b, inset), which bulges vertically to accommodate the distortion of the Ta lattice, and forms truncated triangles located directly above the David-stars (marked red, Fig. 1b, inset). The in-plane CDW charge modulation *δρ* can be described by a set of complex order parameters, 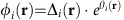, such that 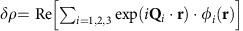. Here **Q**_*i*_ (*i*=1, 2, 3) are the three in-plane reciprocal lattice vectors associated with the 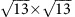 supermodulation, and *θ*_*i*_(**r**) and Δ_*i*_(**r**) represent the phase and amplitude of the CDW charge order, respectively. The regular triangular superlattice seen in Fig. 1b indicates a ground state with uniform *ϕ*_*i*_ in 1T-TaS_2_.

The insulating ground state makes drastic transition to a mosaic state when subjected to a voltage pulse applied across the tip-sample junction at low temperatures. A patch of such pulse-generated mosaic state is presented in Fig. 1c. Inside the patch the originally homogeneous CCDW superlattice disintegrates into nanometre-sized domains separated by well-defined domain wall textures. Within the domains the commensurate David-star configuration is strictly preserved (Fig. 1c, inset), whereas the phase of the CDW order *θ*_*i*_(**r**) undergoes abrupt change across the domain walls, which we shall discuss later.

A detailed study of the pulse parameter is summarized in Supplementary Fig. 1. Empirically, both positive and negative pulses are capable of triggering the transition (Supplementary Fig. 2), and higher pulse voltages tend to create MM states on a larger area, ranging from tens of nanometres to sub-micrometres in diameter (Supplementary Fig. 3). Such mosaic phase is distinctly different from the nearly commensurate CDW phase existing at higher temperatures (refs 9, 35 and Supplementary Fig. 4), but shows strong similarity to the supercooled nearly commensurate CDW state at low temperatures (Supplementary Fig. 5).

**Figure 2:**
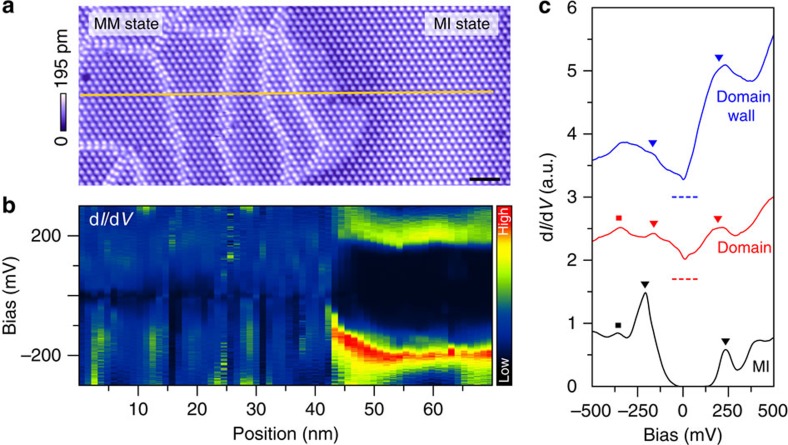
Electronic structure of the MM state. (**a**) STM topography of the MM state interfaced with MI state. Scale bar, 5 nm. Image was recorded under tunnelling condition *V*_t_=0.3 V and *I*_t_=0.1 nA. (**b**) Differential conductance (d*I*/d*V*) as a function of sample bias (vertical axis) and distance (horizontal axis) measured along the yellow line in **a**. (**c**) Spatially averaged d*I*/d*V* spectra acquired in MI state (black), MM domains (red) and domain walls (blue). Curves are vertically shifted for clarity. The squares signify the spectral peaks corresponding to the submanifolds associated with the CCDW formation^42^. The triangles label the position of the Hubbard bands in MI state (black), or the edges of the V-shaped DOS suppression in MM state, which are reminiscent of the Hubbard bands (red and blue).

The electronic structure of the new mosaic state is fundamentally different from that of the original insulating ground state. Whereas the pristine CCDW ground state is an insulator featuring a 430-meV energy gap (Fig. 2c, black), the mosaic state is of a metallic nature with finite local density of states (DOS) around the Fermi level both inside the domains (Fig. 2c, red) and on the domain walls (Fig. 2c, blue). Such a distinction is clearly captured by the differential conductance (d*I*/d*V*) spectral waterfall acquired across a MM–insulator interface shown in Fig. 2b, where a sharp metal–insulator transition occurs within a superlattice unit cell. The transition from MM to insulator is accompanied by prominent deformation of the energy bands on the insulator side, which we attribute to the combined space charging and tip-induced band-bending effect similar to that at a semiconductor–metal interface (Supplementary Figs 6 and 7).

### Metastable nature of the MM phase

**Figure 3:**
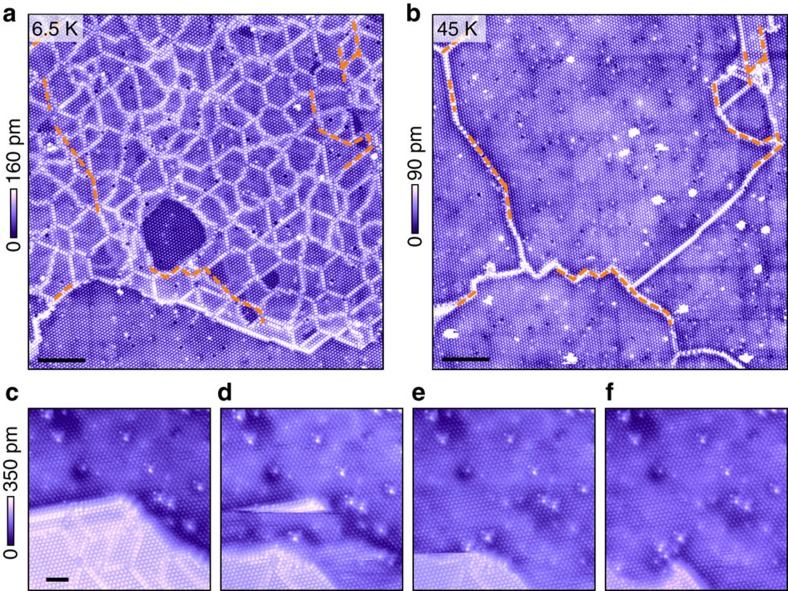
The metastable nature of the MM state. (**a**) Low-temperature STM topography of the MM state recorded at *T*=6.5 K. Scale bar, 20 nm. (**b**) Topographical image taken over the same area as in **a**, but at an elevated temperature of *T*=46 K. Scale bar, 20 nm. The ramping rate of temperature was kept under 0.2 K min^−1^. Part of the sparse residual boundaries (marked by red dash lines) is inherited from original domain walls in the low-temperature MM state as shown in **a**. (**c**–**f**) An MM patch gradually converted to MI state by repeated scanning at *T*=30 K. Scale bar, 5 nm. **c**–**f** are in chronological order, and the data were taken over a 6-min period. STM scanning condition: *V*_t_=0.15 V and *I*_t_=0.01 nA.

Even though the MM state appears stable at low temperatures, we find that the state is in fact metastable in nature. Figure 3a displays a typical pulse-induced MM patch surrounded by the pristine insulating state. No change of the MM patch was observed after weeks of intensive imaging and spectroscopic measurements at *T*=6.5 K. On increasing the temperature, however, the MM patch becomes unstable and the domain structure melts away. Figure 3b display the same area of the sample surface as shown in Fig. 3a, but at an elevated temperature of *T*=46 K. The dense aggregation of domain walls dissolves, leaving an ordered CCDW superlattice decorated with sparse boundary lines, part of which traces the low-temperature domain walls (Fig. 3b, dashed lines). Meanwhile, the insulating behaviour fully recovers over the entire surface except on those boundary lines. Cooling down the sample again does not bring back the MM state, and the insulating state (with boundary lines) persists to low temperatures. Such hysteretic behaviour unambiguously demonstrates that the MM state is metastable. The metastable nature of the MM state is further corroborated by the state's fragility at elevated temperatures: the border of the metallic state gradually recedes when perturbed by repeated scanning of an STM tip at *T*=30 K (tunnelling voltage and current is *V*_t_=150 mV and *I*_t_=10 pA, respectively) (Fig. 3c–f). Finally, we note that the MM phase observed in our experiment may be intimately linked to the metastable metallic states induced by various macroscopic techniques, including both optical excitation^24^ and carrier injection^25^,^28^,^29^,^30^, from the ground state of 1T-TaS_2_. Our STM study may therefore provide crucial microscopic understanding of those phases for the first time.

### Phase configuration of the MM domains

**Figure 4:**
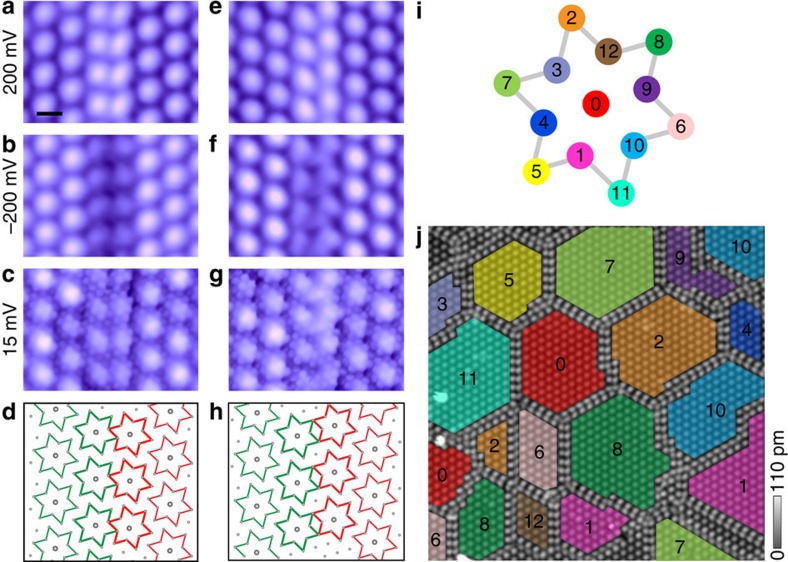
In-plane domain/phase configuration of the CCDW order in the MM phase. (**a**–**c**,**e**–**g**) STM topography two common types of CDW domain walls recorded at varying sample biases. Scale bar, 1 nm. The atomically resolved images at low bias enabled us to establish the structure model of both types of domain walls (**d**,**h**). (**i**) David-star of Ta atoms in the unit cell of the CCDW superlattice. The atoms are numbered following the convention described in ref. 36. Each number represents 1 of the 12 possible relative translations (and corresponding phase differences) of the CCDW superlattices in neighbouring domains. Two consecutive translations can be represented by the difference of the two numbers. (**j**) Topography of an MM state showing multiple domains. The phase of each mosaic domains (relative to the domain **0**) are determined from analysis of domain walls using procedures similar to **a**. The phases are coded by the numbers defined in **i** (see text).

A key feature of the MM state is the constant phase of the order parameter *θ*_*i*_ inside each domain, and abrupt change in *θ*_*i*_ across the domain walls. Shown in Fig. 4a–c,e–g are two most common types of domain wall observed in the MM state. The atomically resolved STM images recorded at low bias (*V*_t_=15 mV) enable us to precisely determine the positions of the David-stars on the domain wall. It turns out that the superlattices of David-stars in the neighbouring domains are shifted against each other by a lattice vector of the underlying two-dimensional atomic crystal, **T**, so that the phase difference between the two domains can be written as


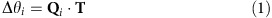


**T** takes the value of −**a**+**b** and −**b** (**a** and **b** are basis vectors of 1T-TaS_2_ two-dimensional crystal) for domain walls shown in Fig. 4a–c,e–g, respectively. As a result two columns of David-stars form the domain wall in an edge-sharing (Fig. 4d) or corner-sharing (Fig. 4h) configuration. No rotation of the David-star triangular lattice was observed on the domain wall, nor was any defect in the underlying atomic crystal.

In fact, surveys of the MM state reveal that equation (1) describes all the domain walls observed in our experiment (Supplementary Figs 8 and 9), which enables us to completely determine the phase configuration of the MM domains. The relative translation **T** in general takes the form **T**=*m***a+***n***b**, where *m* and *n* take integer values such that a **T** sitting at the central Ta atom runs through 12 other Ta on the same David-star (Fig. 4i). There are therefore 12 possible types of domain walls with associated phase difference (*Δθ*_1_, *Δθ*_2_, *Δθ*_3_)=2*π*(3*m*+*n*, −4*m*+3*n*, *m*−4*n*)/13 (Supplementary Fig. 8). Equivalently, the 12 possible **T** can be labelled by the Ta atom that characterizes the translation (Fig. 4i). Here the Ta atoms (and therefore the **T**) are numbered **0** … **12** following the convention adopted in ref. 36, which has the added advantage that two consecutive translations are represented by the difference of the two numbers. Armed with above analysis, we are able to completely delineate the phase configuration of an MM state, such as the one shown in Fig. 4j. Here the phase of all the domains is referenced to the domain **0**, and the relative phase shift between two domains is readily unscrambled by taking the difference of their domain numbers. Finally, we note that out of the 12 possibilities only 4 types of domain walls are observed in our experiment, with varying frequency of occurrence (Supplementary Fig. 9), indicating subtle differences in energy associated with each type of domain walls.

### CCDW stacking order and the electronic structure

**Figure 5:**
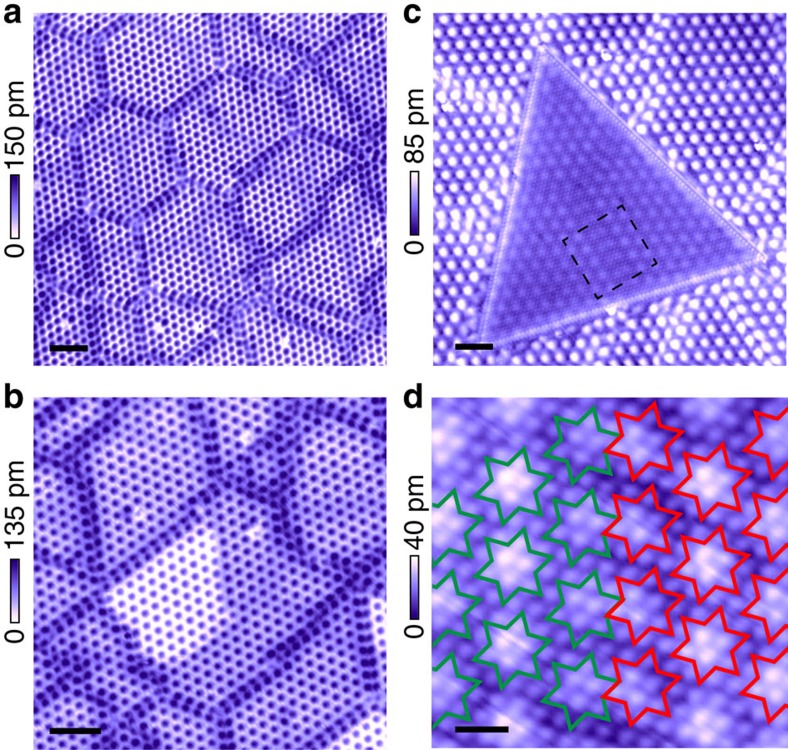
Altered stacking order of the CCDW superlattice in the MM state. (**a**) STM topography of a 50 × 50-nm MM area. STM imaging condition: *V*_t_=0.2 V and *I*_t_=0.1 nA. Apart from the well-defined domain walls in the top layer, domain walls in the layer underneath are clearly resolved as networks of random filamentary features. Here an inverted colour scale is adopted for better contrast. Scale bar, 5 nm. (**b**) An MI patch (bright region) surrounded by MM domains in the MM state. The patch is encircled by domain walls in either the top layer or the second layer. STM imaging condition: *V*_t_=0.3 V and *I*_t_=0.02 nA. Scale bar, 5 nm. (**c**) A triangular patch of H-phase TaS_2_ accidently created in 1T-TaS_2_ by a voltage pulse. The H-phase is induced only on the topmost atomic layer, and the flat surface enabled us to see through the top layer and to observe the 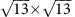 CCDW superlattice as well as domain walls in the layer underneath^15^,^18^. Scale bar, 3 nm. (**d**) Zoomed-in STM image of the area marked by black dashed square in **c** showing atomically resolved domain wall structure in the second layer. The domain wall has a type **2** configuration. Scale bar, 8 Å.

The domain and associated phase structure distinguishes the MM state from its parent MI state, even though the two states share the same CCDW superlattice. There are two main points to notice. First, the MI state is parasitic to the CCDW^27^, rather than a requisite as suggested by some of the previous works^37^,^38^. Second, phase shift of the CCDW order in one atomic layer implies a shifted CCDW superlattice relative to other layers. In fact, not only do we see phase shifts in the topmost layer, clear signature of random domain wall networks are also observed in the second layer (Fig. 5a). One example of such domain wall is shown in Fig. 5c, where a flat monolayer H-TaS_2_ patch (also induced by the same voltage pulse) enables us to see through the top layer and resolve the domain wall's atomic structure. We find that the domain wall in the second layer also corresponds to a phase shift described by equation (1) (Fig. 5d). The presence of randomly distributed domains and phases in two adjacent layers therefore incurs a randomized stacking of the CCDW superlattices.

We are now poised to address the central question: how does the MI state emerge from CCDW, against competing metallic state in 1T-TaS_2_? An important clue comes from rare occurrences such as the one shown in Fig. 5b, where a small insulating patch appears inside metallic random MM domains. Close examination reveals that the insulating patch is surrounded partly by the topmost-layer domain walls, and partly by the domain wall in the second layer. Such a domain wall configuration leads to a distinct stacking order of CCDW superlattice that restores the insulating state inside the patch, in contrast to the surrounding domains still in the MM state. The existence of uniform insulating patch with sharp boundaries also rules out carrier doping and disorder potential^39^,^40^ as the cause of the insulator–metal transition. Our observations therefore point to interlayer stacking as a decisive factor in determining the electronic structure of 1T-TaS_2_ ground state.

**Figure 6:**
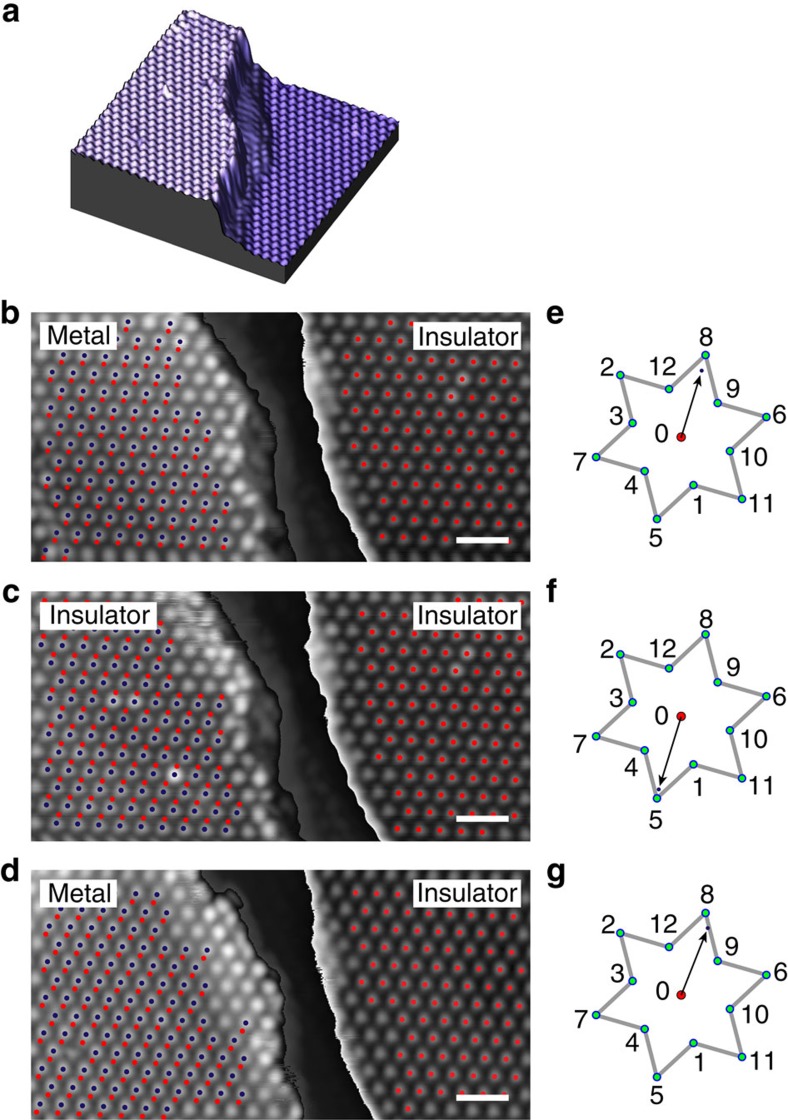
Reversible switching of metal–insulator domain and the corresponding stacking order. (**a**) Three-dimensional rendering of a 30 × 30-nm STM image of a monolayer terrace on 1T-TaS_2_ surface. The height of the terrace is 5.93 Å. Image was recorded at 6.5 K. (**b**–**d**) STM topography of the same area shown in **a**. The upper terrace was switched from metallic state (**b**) to insulating state (**c**) and back to metallic state (**d**), by gentle voltage pulses (∼1.5–2 V, 50-ms duration). All other domains were kept intact during the pulses. The red (blue) dot array marks the CCDW lattice on the lower (upper) terrace, respectively. The relative CCDW lattice shift can be extracted by extrapolating the position of the array on the lower terrace to the upper terrace, and projecting the relative displacement onto a CCDW unit cell. Scale bars, 3 nm. (**e**–**g**) The CCDW lattice shift between the upper and lower terraces extracted from the analysis shown in **b**–**d**, respectively. The shifts (arrows and black dots) are projected relative to **0** onto the CCDW unit cell (David-star). Reversible switching of the metal–insulator domain is directly linked to the reversible switching of the CCDW stacking order.

Unambiguous evidence directly linking the electronic structure and the CCDW stacking order comes from a reversible, tip-induced metal-to-insulator transition on a monolayer terrace on 1T-TaS_2_ surface as shown in Fig. 6a. We were able to use gentle voltage pulses to reversibly switch the upper terrace from metallic (Fig. 6b) to insulating (Fig. 6c) and back to metallic (Fig. 6d) state at 6.5 K, while keeping the lower terrace intact. The CCDW lattice shift was carefully determined for each of the three states. It turns out that switching from metallic to insulating state corresponds to a CCDW stacking shift of **8**→**5** (Fig. 6e,f), following the notation defined in Fig. 4i, with the lower terrace taken to be the reference (**0**). Such correspondence is reversible and reproducible such that the metallic nature of the upper terrace is recovered when the stacking sequence is switched back to **8** (Fig. 6g). Our experiment therefore establish the direct link between the CCDW stacking order and the electronic structure of the domains. Finally we note that the stacking orders of **0**→**8** and **0**→**5** produce metallic and insulating states, respectively, in contradiction with the seeming degeneracy of the two stacking orders. Such degeneracy is however lifted if the CCDW unit cell deviates from a perfect David-star (slight distortions indeed appear in our *ab initio* calculations), or if there is a domain boundary in the lower layer under the step edge, which gives an additional lattice shift. Next-nearest-neighbour coupling with the third layer may also be able to lift the degeneracy.

## Discussion

The importance of the interlayer stacking can be understood from a three-dimensional Hubbard model with intra- and interlayer hopping taken into account. The one-band Hamiltonian of 1T-TaS_2_ CCDW ground state can be written as:


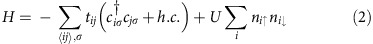


where *t*_*ij*_ is the effective hopping between the David-stars and *U* the on-site Coulomb repulsion on one David-star. Because of the flat pancake shape of the David-star, the interlayer distance of the CCDW superlattice (5.9 Å) is significantly shorter than the in-plane separation between the centres of neighbouring David-stars (12.1 Å; ref. 7). Various experimental and theoretical studies^33^,^34^,^41^,^42^ have suggested the importance of interlayer coupling. Indeed, our angle-resolved photoemission spectroscopy measurements on the pristine crystal reveals a bandwidth of *W*∼50 meV for the lower Hubbard band in both **k**_*z*_ and **k**_||_ direction (Supplementary Fig. 10). This observation indicates that the effective out-of-plane hopping factor *t*_‖_ is comparable to its in-plane counterpart *t*_||_. We note that *U*/*W*∼8 for pristine 1T-TaS_2_, a typical value for a MI ground state.

A MI to metal transition occurs on increasing the bandwidth *W* with respect to the Hubbard *U*, where *W* is determined by the effective hopping factors *t*_‖_ and *t*_||_ (with coordination number taken into account). As the stacking order of the CCDW is varied, the variation in the separation between the David-stars in neighbouring layer (as well as coordination number) could bring drastic change in *t*_‖_. We speculate that in certain stacking configurations, the bandwidth *W* is driven beyond certain critical value, and the MI insulator to metal transition becomes a possibility. Such a speculation is supported by recent density functional theory calculations suggesting that with an altered interlayer stacking sequence, *t*_‖_ may experience an order of magnitude increase, and brings *W* to the same order of magnitude as *U* (ref. 34).

The rare occurrence of the insulating domain among the randomly stacked MM domains (Fig. 5b) implies that only a small number of stacking order yields insulating states with the rest metallic in nature. The exact interlayer stacking order of the various states, however, is not directly determined due to an intrinsic limitation of STM; tunnelling microscopy could only resolve the atomic structure of the topmost layer. Here we point out that even the stacking order in the pristine CCDW ground state remains elusive^36^,^43^,^44^,^45^,^46^, and we call for further experimental and theoretical work to clarify the exact stacking order of the various charge-ordered states. Finally we note that an alternative scenario, where orbital order, instead of electron–electron interaction, dominates the electronic structure of the CCDW phase, has been proposed recently^34^. In this scenario, the stacking order (which determines the orbital order) would also dictates the electronic state of 1T-TaS_2_. However, calculations within this framework invariably predict a dispersive band (therefore a finite DOS) crossing Fermi level in the out-of-plane direction in the pristine insulating state. The lack of such dispersive band in angle-resolved photoemission spectroscopy measurements (Supplementary Fig. 10) and the observation of a fully gapped DOS near Fermi level (Fig. 2c, black curve) are evidences against the orbital-order scenario. It remains to be seen whether theories based on orbital order could reconcile these conflictions and provide a complete, quantitative description of the various CDW states observed in 1T-TaS_2_.

In summary, we studied a mosaic, metallic state induced from the Mott-insulating CCDW phase in 1T-TaS_2_ by voltage pulses. The mosaic phase features a fragmented in-plane phase distribution of the CDW order parameter, and exhibits metallic behaviour. We discovered that the relative phase shift between adjacent layers leads to an altered local stacking order, which dictates whether the resulting structure is a MI or a metal. Our results therefore shed fresh light to the origin of the insulating ground state of 1T-TaS_2_, and uncover the interlayer coupling as the root. In addition, the MM phase bears strong similarities to the metastable metallic states induced by various external perturbations such as ultra-fast laser pulses^24^ and current excitations^25^,^28^,^29^,^30^. Our study may provide a microscopic understanding for those novel phases that are of importance in practical applications.

## Methods

### Sample preparation and STM measurements

High-quality 1T-TaS_2_ single crystals were grown using a standard chemical vapour transport method. The samples were cleaved in high vacuum at room temperature, and subsequently cooled down for STM measurements. STM experiments were performed in a commercial low-temperature STM (Createc Fischer & Co. GmbH) operated in ultrahigh vacuum. Electrochemically etched polycrystalline tungsten tips calibrated on clean Au(111) surfaces were used for all our STM measurements. Typical tip-calibration data are shown in Supplementary Fig. 11. The STM topography was taken in the constant-current mode, and the d*I*/d*V* spectra were collected using a standard lock-in technique, with a modulation frequency of 789.1 Hz. Before we opened the feedback loop to apply voltage pulses, the tip was parked above the sample surface under typical tunnelling condition of *V*_t_=0.3–0.5 V and *I*_t_=0.3–1 nA. The pulse duration was fixed at 50 ms.

### Calculation of the energy associated with various stacking order

Fully relaxed David-star geometry for single and bilayer 1T-TaS_2_ was obtained from density functional theory calculations using Quantum Espresso Code^47^. We adopted the PBE (Perdew-Burke-Ernzerhof) generalized gradient approximation^48^ for the exchange-correlation functional, the norm-conserving pseudo-potential^49^, and an energy cutoff of 55 Ry and 6 × 6 *k*-points for the supercell. In the relaxed bilayer 1T-TaS_2_, the centre of David-star in the top layer is found to shift laterally by **a** relative to the centre in the bottom layer. For trilayer 1T-TaS_2_, the binding energy is calculated for two cases: (1) the centre of the David-star in the third (bottom) layer shifting laterally by 2**a** with respect to the centre in the first (top) layer, that is, ABC stacking; and (2) the centre in the third layer aligning with that in the topmost layer, that is, ABA stacking. We computed the energy associated with various stacking orders as the David-star in one layer is displaced relative to that in other layers, and found the energy difference is on the order of 6.4 meV.

## Additional information

**How to cite this article:** Ma, L. *et al*. A metallic mosaic phase and the origin of Mott-insulating state in 1T-TaS_2_. *Nat. Commun.* 7:10956 doi: 10.1038/ncomms10956 (2016).

## Supplementary Material

Supplementary Information Supplementary Figures 1-11 and Supplementary References
