# Supplementary material for: A metallic mosaic phase and the origin of Mott-insulating state in 1T-TaS2
Source: Nat Commun. 2016 Mar 10;7:10956. doi: 10.1038/ncomms10956 (PMC4792954; doi:10.1038/ncomms10956)
Supplement: Supplementary Information — Supplementary Figures 1-11 and Supplementary References [file ncomms10956-s1.pdf]

Supplementary Figures

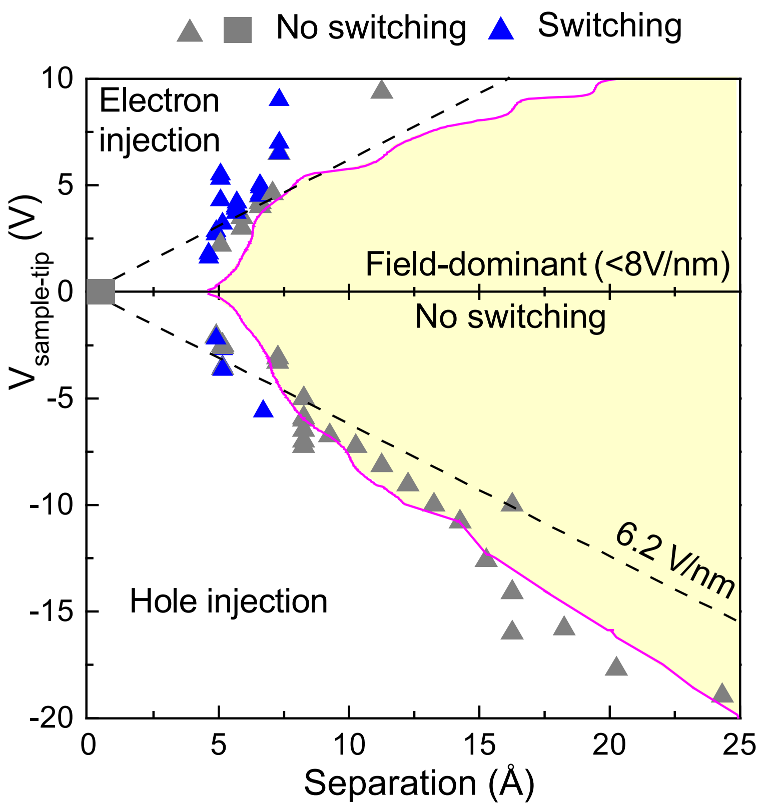

Supplementary Figure 1 | Statistics of experimental attempts of inducing phase-switching with controlled tip-sample voltage and separation. The magenta line corresponds to the tunnelling condition under which the tunnelling current between the sample and tip is fixed at 1nA. The enclosed parameter space (marked yellow) therefore corresponds to tunnelling with current less than 1nA. We found that continued scanning in yellow region did not produce any phase switching, even if the electric field is increased up to 8 V/nm. (The grey triangles denote individual pulses that did not produce phase switching either.) Meanwhile, pulses with similar electric field but smaller tip-sample separation (*i.e.* larger tunnelling/emission current; up to 20 nA in our experiment) can trigger the switching (blue triangles). We thus conclude that a pure electric field (without current) cannot switch the phase. We further studied the effect of a pure current injection (up to 100 uA) through the point contact between STM tip and sample without the presence of a large electric field (grey square), and didn't observe phase switching. This rules out ohmic joule heating as the driving force of the phase switching. Because both high electric field and current are required to induce the phase switching, we speculate that sudden local heating by hot electron/holes, followed by quenching, causes the switching[1].

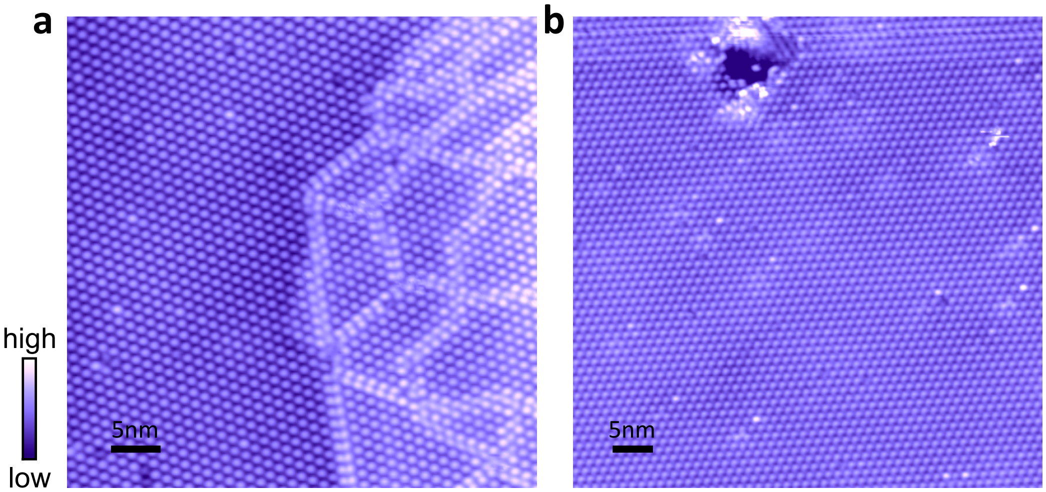

Supplementary Figure 2 | MM phase induced by negative voltage pulse and the lack of phase switching from mechanical damage. a, STM image of a MM patch induced by a −3.6 V pulse. **b,** STM image after mechanically crashing the STM tip onto the sample. MI to MM transition was not observed.

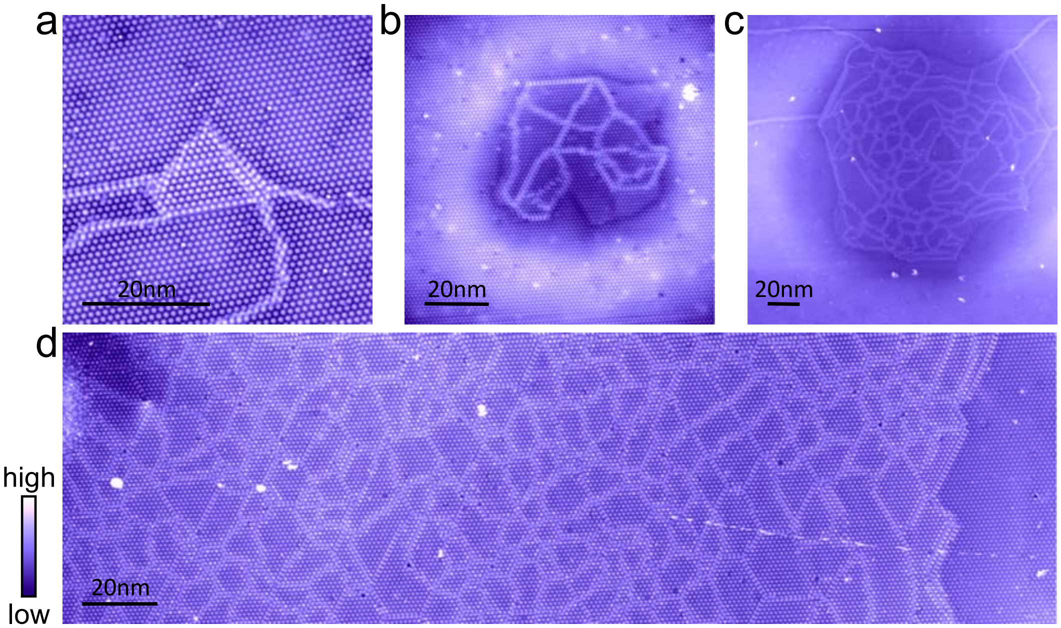

Supplementary Figure 3 | Controlling the size of the MM patches by pulse voltage. a-d, STM images of MM patches created by voltages pulses of 1.6, 2.7, 3.2 and 9.0 V, respectively. The diameters of the MM patches in **a-d** are about 15 nm, 50 nm, 130 nm, 500 nm (only part of the patch is shown here), respectively.

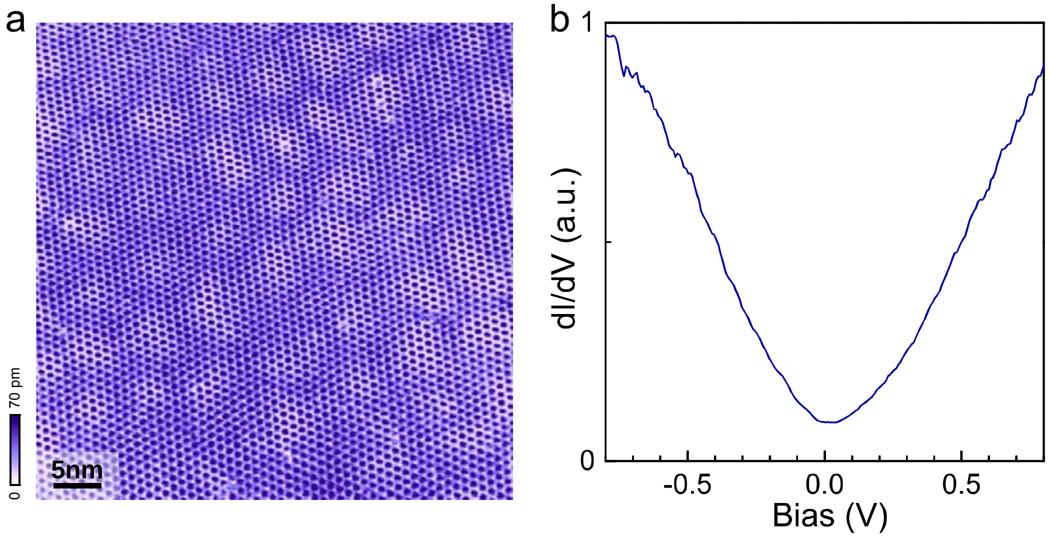

Supplementary Figure 4 | Nearly commensurate CDW (NCCDW) phase of 1T-TaS_2 at room temperature. a, STM image of NCCDW phase at 300K. CCDW domains are separated by incommensurate domain wall networks. The CDW phase order parameters are different between domains while the amplitude was smoothly modulated from domain centre to domain wall. **b,** dI/dV spectrum acquired on surface of NCCDW phase at 300K. The induced MM phase is similar to NCCDW phase. They both are conductive and exhibiting a domain-like pattern. The average domain size of MM phase is 80 nm^2, about the same with that of NCCDW phase[2] near the NC to C transition point (200K). The difference is NCCDW phase has distorted Kagome lattice domains[3] (or so called corner sharing hexagonal domains) with broadened domain walls while complex domain distribution with sharp domain walls in MM phase.

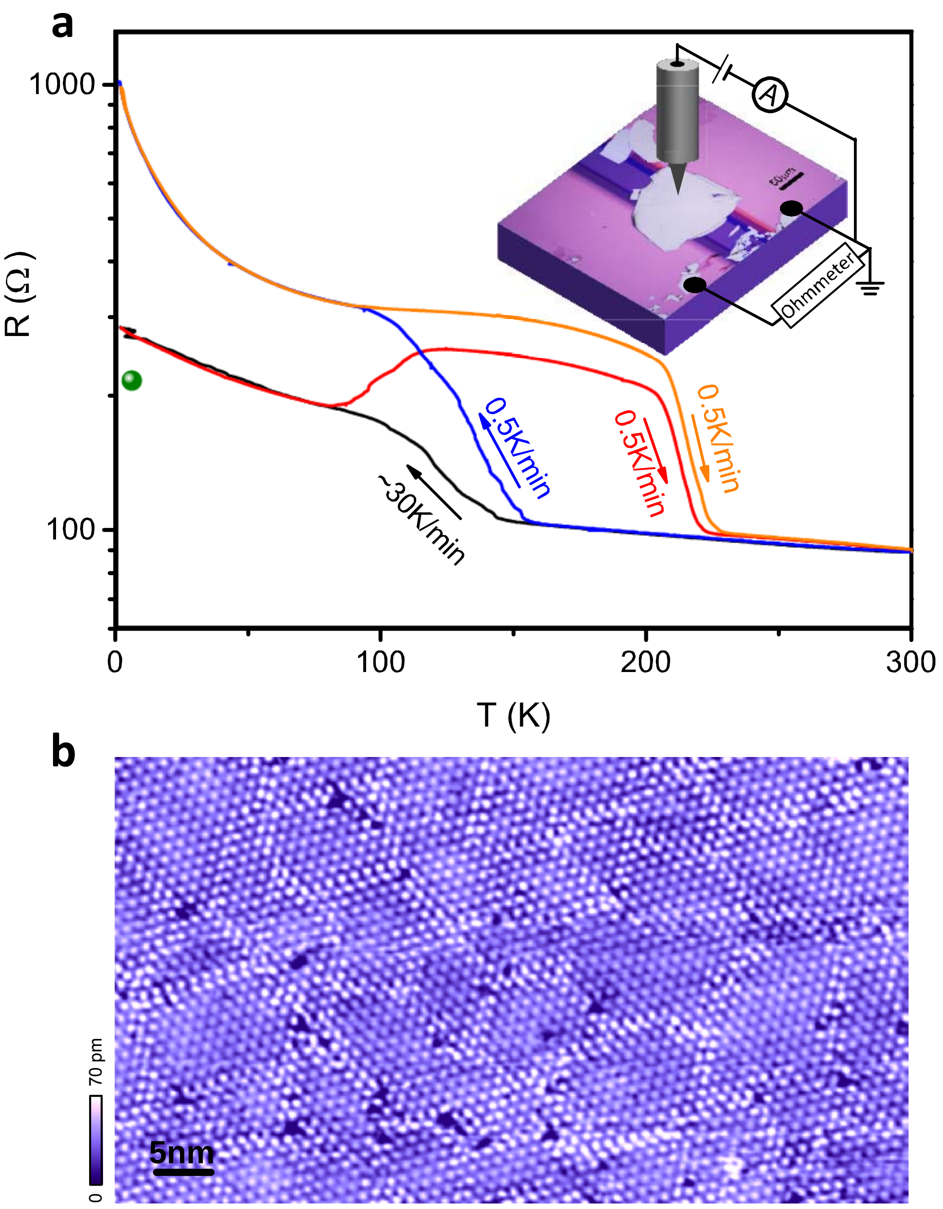

Supplementary Figure 5 | Transport and STM characterisation of the super-cooled NCCDW phase. a, Resistance as a function of temperature of a 1T-TaS_2 thin flake under various cool-down and warm-up rate. The CCDW state is developed at low temperature under a low cool-down rate (blue curve), whereas under a high cool-down rate a super-cooled NCCDW state is observed[4] (black curve). **b,** STM image of the super-cooled NCCDW state obtained on the same device as shown in the inset of **a** (imaging condition: $V_t = 1$ V and $I_t = 60$ pA). The super-cooled state was realized by quenching the device from room temperature to liquid helium temperature (with an effective cooling rate of ~ 70 K/min). The low-temperature sample resistance is shown in **a** (green dot), indicating that the sample is indeed in the super-cooled NCCDW state. The topography of the super-cooled NCCDW phase shows randomly distributed domains, which are similar to those in the pulse-induced MM phase.

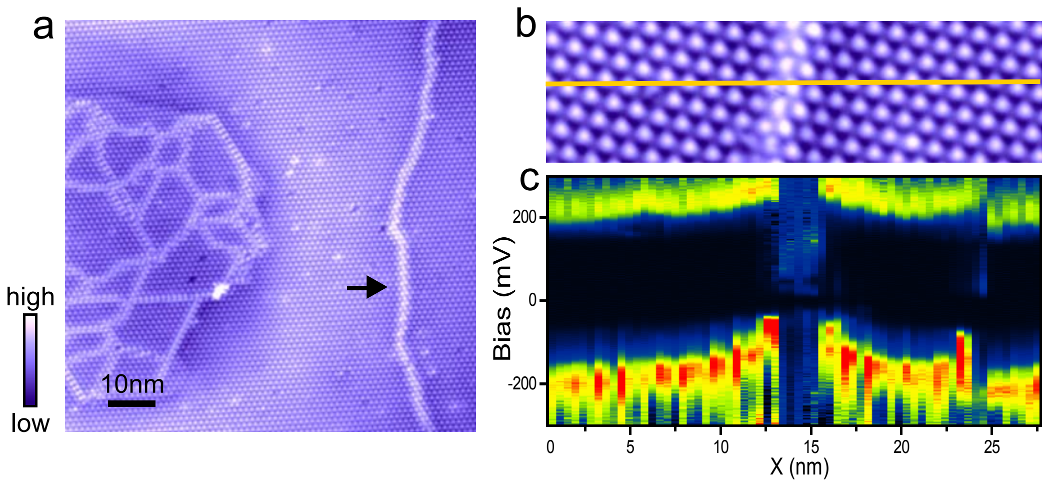

Supplementary Figure 6 | A conductive domain boundary in insulating CCDW phase induced by voltage pulse. a, STM image of a single domain boundary (indicated by the black arrow) and the MM phase, both of which are induced by a voltage pulse. Such domain boundaries inside of CCDW phase are occasionally observed after voltage pulses. The length of the boundary is typically on the order of ~ 100 nm. **b,** Zoomed-in STM image of the domain boundary in **a**. The boundary also corresponds to a phase shift in the phase of the CDW order parameter. **c,** dI/dV spectrum along the orange line in **b**. The Mott-Hubbard gap disappears at the domain boundary, and the boundary exhibits a metallic behaviour.

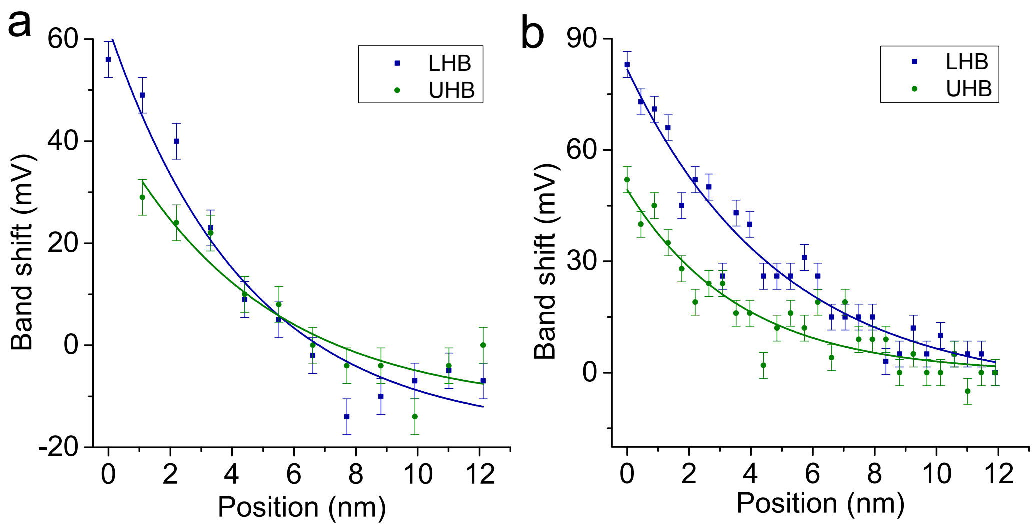

Supplementary Figure 7 | Band bending at MM-MI interface and at conductive boundary in CCDW phase. a, Bending of the upper and lower bands near the interface between the MM phase and MI phase. The position is measured relative to the interface where the gap closes. Error bars represent measurement uncertainties. **b,** Bending of the upper and lower bands near the conductive boundary in insulating CCDW phase discussed in Supplementary Figure 6. Error bars represent measurement uncertainties. Lines are exponential fits to the data sets. The band bending effect in a Mott insulator has been studied theoretically by Takashi Oka et al. (ref. 5). It was shown that the band bending in Mott insulator is also described by Poisson's equation, which is qualitatively not different from that in conventional band insulators. The exponential decay of the bending effect observed at the domain interface of 1T-TaS_2 therefore fits the behaviour expected in both types of insulators. From the exponential fit of the band shifts, we obtained a decay length of ~ 4 nm. Such a length scale of course has different interpretations when different types of insulators are assumed. If a band insulator is assumed, we were able to estimate a space charging region width $W_{SCR} \approx 5$ nm based on the model described in ref. 6. Here a Schottky barrier height of 0.08 eV was estimated from band shift observed in our tunnelling spectra, and the relative permittivity of 10 and a free carrier concentration of $\sim 4 \times 10^{18}$ cm^{-3} were obtained from ref. 7, respectively. Such a W_{SCR} is on the same order of magnitude as the measured decay length. It would be ideal if a similar estimation could be performed in the case of Mott insulator. But unfortunately such an estimation is not possible because important parameters such as electron density-potential relation are not currently available.

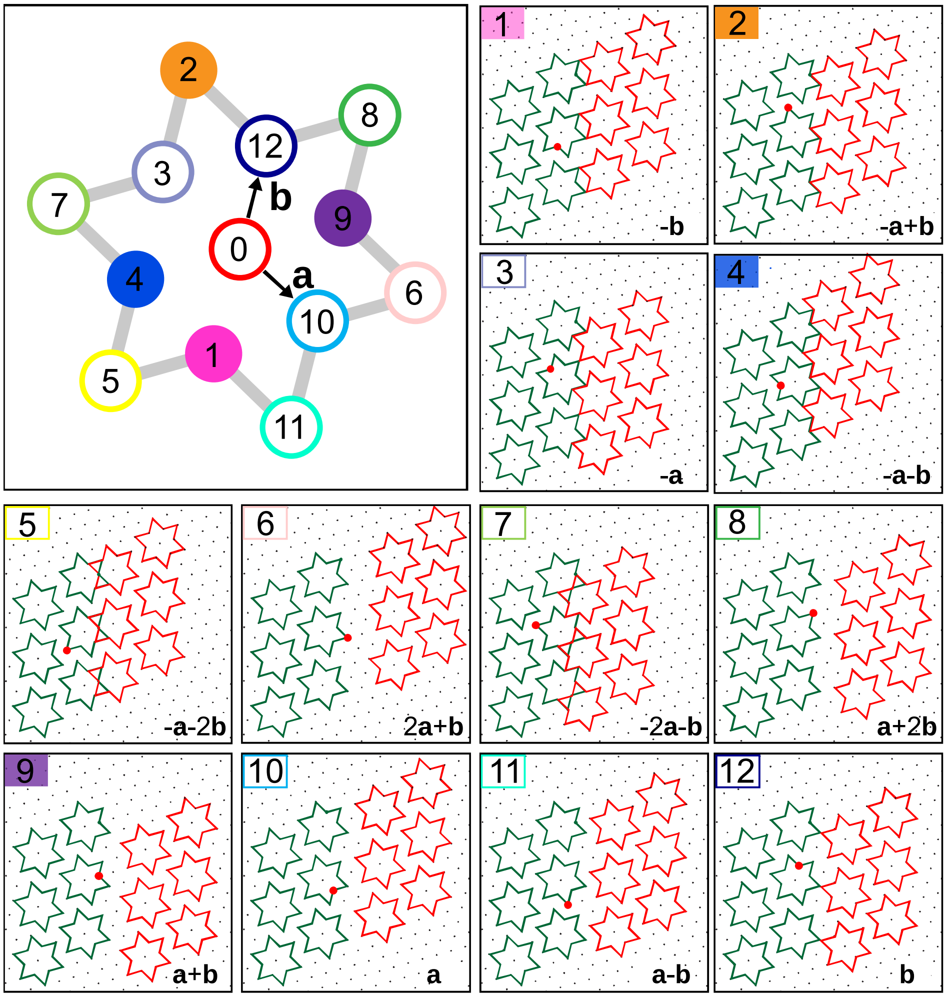

Supplementary Figure 8 | Twelve possible boundary configurations between adjacent CCDW domains. Upper-left panel: the thirteen Ta atoms in a David-star unit cell of the CCDW phase of 1T-TaS_2. **a** and **b** are unit vectors of the underlying atomic lattice. (unit vectors of the CCDW superlattice can then be written as $\mathbf{A} = 4\mathbf{a} + \mathbf{b}$ and $\mathbf{B} = 3\mathbf{b} - \mathbf{a}$.) Panel 1-12 show the twelve possible domain wall configurations. Assuming the underlying atomic lattice remain intact and there is no relative rotation of the CCDW order, the twelve domain wall configurations shown here exhaust all possibilities. Each possible configuration can be obtained by translating the David-star pattern (red) relative to that of the adjacent domain (green), so that the centre of the David-star, up to a CCDW superlattice vector, coincides with one of the twelve surrounding Ta atoms (marked by the red dot in panel 1-12; number of the atom shown on the upper-left corner). Here we adopt the convention used in ref. 8 to number the Ta atoms, which has the advantage that two consecutive translation can be represented by the difference of the two numbers. The corresponding shift of the David star centre, $m\mathbf{a} + n\mathbf{b}$ (m and n take integer values), is indicated in each panel. Each domain wall configuration, therefore, is characterized by the shift in phase of the CDW order parameter, $(\Delta\theta_1, \Delta\theta_2, \Delta\theta_3) = 2\pi(\frac{3m+n}{13}, \frac{-4m+3n}{13}, \frac{m-4n}{13})$. The numbers on solid colour indicate the domain wall configurations that are observed in the experiment (Supplementary Figure 7).

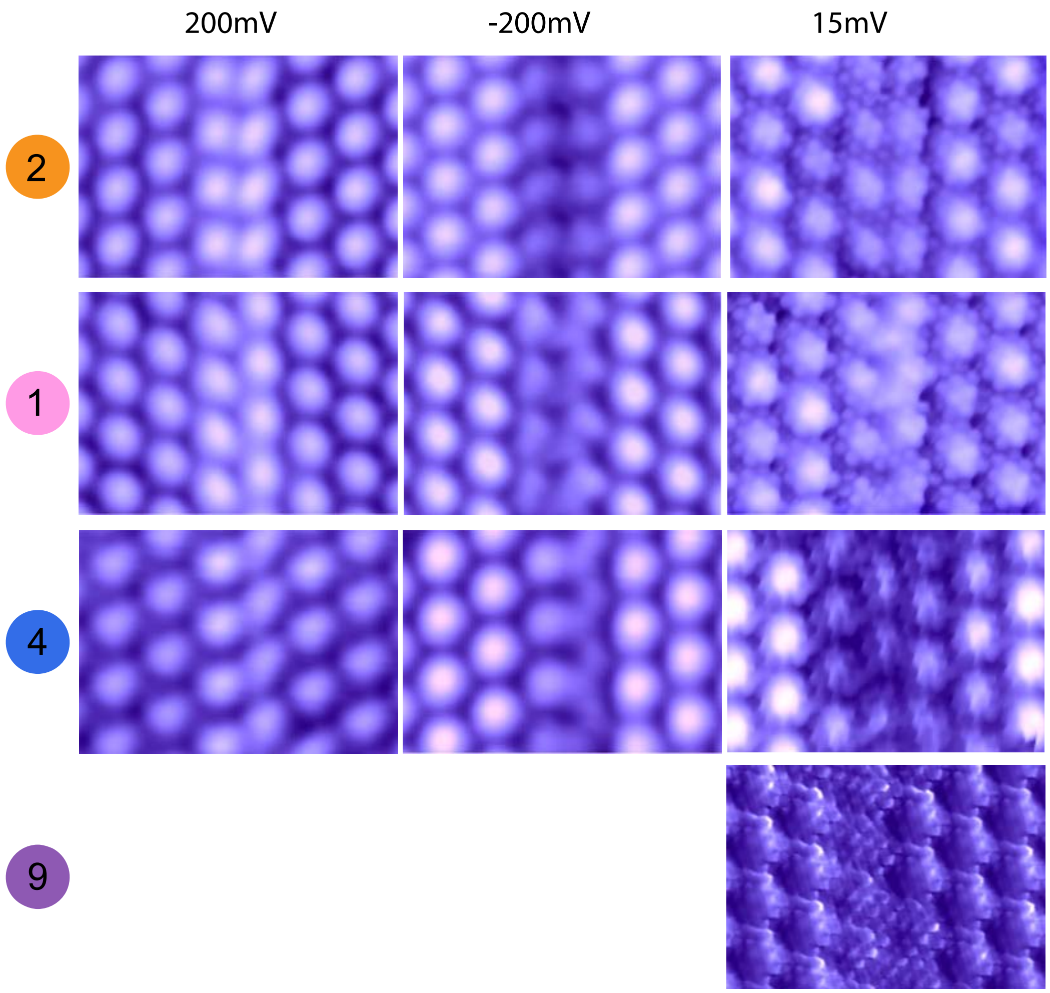

Supplementary Figure 9 | Four types of domain walls observed in our experiments. STM images of all four types of domain walls observed in our experiments under sample bias of 200 mV, -200 mV and 15 mV, respectively. All the experimentally observed domain walls are described by the atomic configurations (**2**, **1**, **4** and **9**, specifically) discussed in Supplementary Figure 8. The first two (**2** and **1**) are two most common types of domain walls seen in the experiment, and the last one (**9**) is rarely seen.

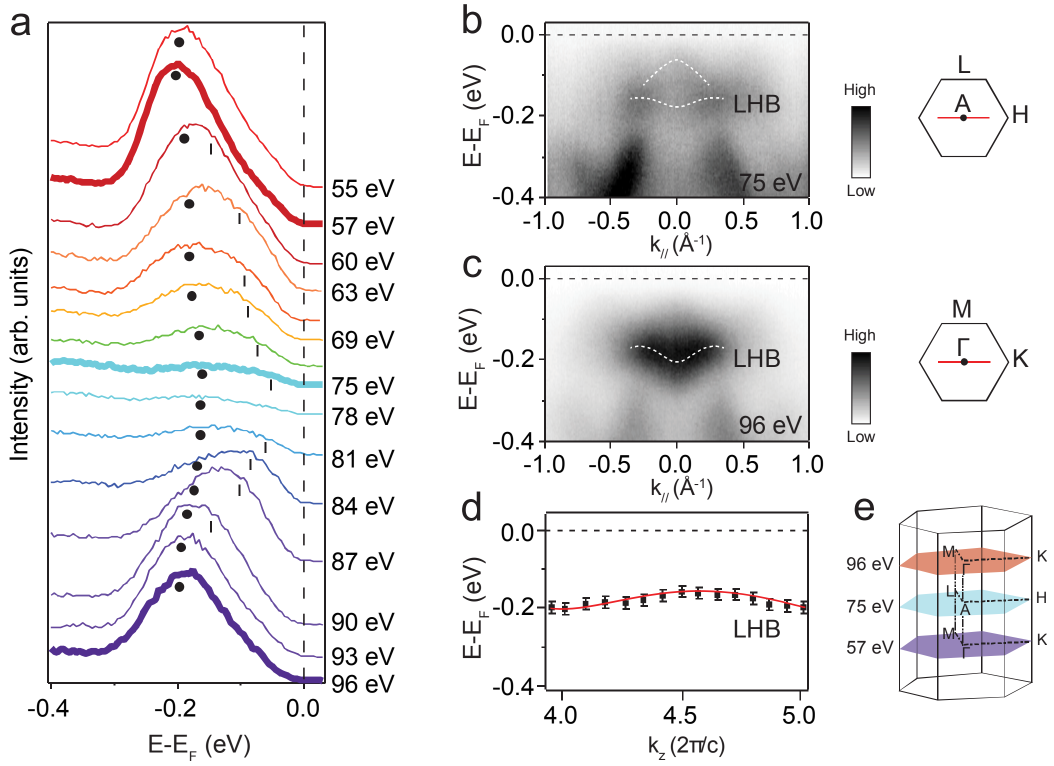

Supplementary Figure 10 | ARPES characterisation of single-crystal 1T-TaS$_2$. a, Normal emission ARPES spectra of 1T-TaS$_2$ taken in the CCDW phase at $T = 30$ K. The photon energy varies from 55 eV to 96 eV. The strong intensity pileup (marked by black circles) at around 200 meV below E_F corresponds to the lower Hubbard band (LHB), and an additional dispersive peak (marked by ticks) appears inside of the Mott-Hubbard gap. Both features agree with those observed in ref. 9. The photon-energy-dependent spectra map out the dispersion of the energy bands in k_z direction, with the purple, blue and red curves corresponding to colour-coded high-symmetry planes in k space shown in **e**. We did not observe energy bands that cross the Fermi level in k_z direction, in contrast to recent predictions based on *ab initio* calculations[10]. **b** and **c**, ARPES spectra of 1T-TaS$_2$ along $\mathrm{A}-\mathrm{H}$ and $\Gamma-\mathrm{K}$ directions (indicated in the 2D Brillouin zone shown on the right), respectively. White dashed lines are guides to the eye showing the dispersion of the LHB along the two directions. From the dispersion we obtain the in-plane bandwidth of the LHB, $w_{\parallel} \approx 50$ meV. **d,** k_z dispersion of the LHB extracted from **a**. Error bars represent the uncertainty of determining band position by a Lorentz-fitting procedure. The bandwidth of LHB along k_z direction, $w_{\perp} \approx 40$ meV, is comparable to the in-plane bandwidth. **e,** Brillouin zone of 1T-TaS$_2$ showing high-symmetry planes and their corresponding photon energy. High-resolution ARPES measurements were performed at the SIS beamline of Swiss Light Source (SLS) equipped with a Scienta R4000 electron analyser. The overall energy resolution was better than 20 meV, and the angular resolution was 0.3 degrees. Samples were cleaved in ultra-high vacuum. ARPES measurements for each sample were carried out within 8 hours, with the sample aging effects carefully monitored.

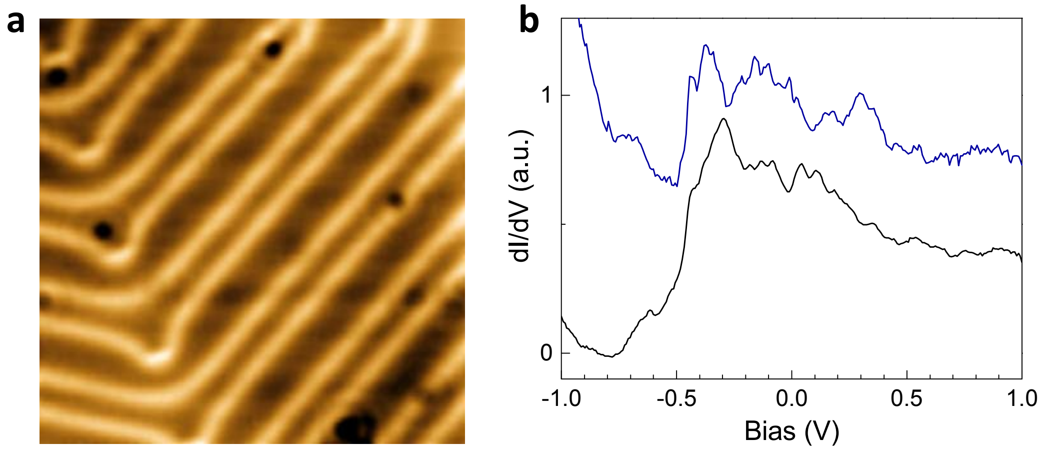

Supplementary Figure 11 | Spectral calibration of the STM tip on Au(111) surface before and after measurements on 1T-TaS$_2$. **a,** Constant-current STM image of Au(111) surface ($V_s = 0.5$ V, I_t =10 pA) showing herringbone reconstruction. **b,** Typical *dI/dV* spectrum measured on Au(111) surface before (black) and after (blue) the STM tip was used to measure 1T-TaS$_2$ samples. All the STM tips used in this study were calibrated on clean Au(111) surface prior to measurement on 1T-TaS$_2$ to ensure that the tips show Au(111) surface state at $V_s = -0.5$ V, similar to that in ref. 11. The tips were checked again on Au(111) surface after the measurement on 1T-TaS$_2$ to confirm that the surface state can be reproduced. This calibration procedure ensures that the tip does not have anomalous spectroscopic features during the measurement on 1T-TaS$_2$.

Supplementary References

1. Wang, H., Lee, J., Dreyer, M. & Barker, B. I. A scanning tunneling microscopy study of a new superstructure around defects created by tip–sample interaction on 2H- $NbSe_2$. *J. Phys. Condens. Matter* **21,** 265005 (2009).
2. Wu, X. L. & Lieber, C. M. Direct observation of growth and melting of the hexagonal-domain charge-density-wave phase in 1T-TaS_2 by scanning tunneling microscopy. *Phys. Rev. Lett.* **64,** 1150–1153 (1990).
3. Spijkerman, A., de Boer, J. L., Meetsma, A., Wiegers, G. A. & van Smaalen, S. X-ray crystal-structure refinement of the nearly commensurate phase of 1T-TaS_2 in (3+2)-dimensional superspace. *Phys. Rev. B* **56,** 13757–13767 (1997).
4. Yoshida, M., Suzuki, R., Zhang, Y., Nakano, M. & Iwasa, Y. Memristive phase switching in two-dimensional crystals. Preprint at http://arxiv.org/abs/1505.04038 (2015)
5. Oka, T. & Nagaosa, N. Interfaces of Correlated Electron Systems: Proposed Mechanism for Colossal Electroresistance. *Phys. Rev. Lett.* **95,** 266403 (2005).
6. Zeghbroeck, B. V. *Principles of Semiconductor Devices and Heterojunctions*. (Prentice Hall PTR, 2007).
7. Bayliss, S. C., Ghorayeb, A. M. & Guy, D. R. P. Thermal and transport evidence for a phase transition in 1T-TaS_2 observed at 282K upon warming. *J. Phys. C Solid State Phys.* **17,** L533 (1984).
8. Nakanishi, K. & Shiba, H. Theory of Three-Dimensional Orderings of Charge-Density Waves in 1T-TaX_2 (X: S, Se). *J. Phys. Soc. Jpn.* **53,** 1103–1113 (1984).
9. Pillo, T. *et al.* Fine structure in high-resolution photoemission spectra of quasi-two-dimensional 1T-TaS_2. *Phys. Rev. B* **64,** 245105 (2001).
10. Ritschel, T. *et al.* Orbital textures and charge density waves in transition metal dichalcogenides. *Nat. Phys.* **11,** 328–331 (2015).
11. Chen, W., Madhavan, V., Jamneala, T. & Crommie, M. F. Scanning tunneling microscopy observation of an electronic superlattice at the surface of clean gold. *Phys. Rev. Lett.* **80,** 1469 (1998).
